# RETRACTION: Best Practices for Managing Malodorous and Infected Wounds in Advanced Cervical Cancer

**DOI:** 10.1111/iwj.70486

**Published:** 2025-04-02

## Abstract

**RETRACTION:** HamidH. A. , LinX. , QinY. K. , AkimA. M. , ZhangL. , WangJ. , WangH. , LiY. , TengX. , ZhangS. , XuH. , and LinX. , “Best Practices for Managing Malodorous and Infected Wounds in Advanced Cervical Cancer,” International Wound Journal 21, no. 2 (2024): e14574, 10.1111/iwj.14574.38379231 PMC10834147

The above article, published online on 01 February 2024, in Wiley Online Library (http://onlinelibrary.wiley.com/), has been retracted by agreement between the journal Editor in Chief, Professor Keith Harding; and John Wiley & Sons Ltd. Following an investigation by the publisher, all parties have concluded that this article was accepted solely on the basis of a compromised peer review process. The editors have therefore decided to retract the article. The authors did not respond to our notice regarding the retraction.

**RETRACTION:** H. A. Hamid , X. Lin , Y. K. Qin , A. M. Akim , L. Zhang , J. Wang , H. Wang , Y. Li , X. Teng , S. Zhang , H. Xu , and X. Lin , “Best Practices for Managing Malodorous and Infected Wounds in Advanced Cervical Cancer,” International Wound Journal 21, no. 2 (2024): e14574, 10.1111/iwj.14574.38379231 PMC10834147

The above article, published online on 01 February 2024, in Wiley Online Library (http://onlinelibrary.wiley.com/), has been retracted by agreement between the journal Editor in Chief, Professor Keith Harding; and John Wiley & Sons Ltd. Following an investigation by the publisher, all parties have concluded that this article was accepted solely on the basis of a compromised peer review process. The editors have therefore decided to retract the article. The authors did not respond to our notice regarding the retraction.

